# Romantic Jealousy, Intimate Partner Violence, and Envy: An Ethnographic Study of Acid Attacks in Cambodia

**DOI:** 10.1177/08862605251318270

**Published:** 2025-05-26

**Authors:** Maurice Eisenbruch

**Affiliations:** 1 Monash University, Clayton, VIC, Australia; 2 University of Melbourne, Parkville, VIC, Australia

**Keywords:** acid attack, gender-based violence, coercive control, romantic jealousy, envy, omens, Buddhist psychotherapy

## Abstract

Acid attacks are generally considered to be a pernicious expression of gender-based violence (GBV) and a global health issue that until recently mainly affected countries in Africa, South and Southeast Asia, and Latin America. However, little is known about the cultural contexts for acid attacks and, in particular, the culture-gender intersect. In Cambodia, the first publicly reported case took place in 1999, and attacks have continued since then. This study aims to identify the cultural construction and meaning of acid attacks from the inside out to provide evidence to guide culturally acceptable interventions. Ethnographic fieldwork was conducted with survivors, their families, and perpetrators in towns and villages across Cambodia, representing 88 cases of acid attacks. Qualitative analysis was conducted to identify the cultural beliefs related to the perceived causes and significance of acid attacks. The “cultural attractors” driving acid attacks are based on Khmer Buddhist beliefs such as karmic links between perpetrator and their target, inherited endowment, character, the Buddhist “triple poison,” zodiacal birth status, astrological incompatibility of a couple, and moral blindness. One group of attacks can be seen as gender-based, either triggered by romantic jealousy or in the context of intimate partner violence. A second group, triggered by envy, is not gender-based and arises as a result of community conflict and inequity. The analysis of conceptual metaphors can enrich our understanding of the complex emotions of romantic jealousy and envy. The cultural lens enriches an intersectoral understanding of violence, including GBV, wherein local Buddhist “cultural attractors” explain the cruelty of perpetrators and the suffering of survivors. Further research can inform the cultural responsiveness of multidisciplinary interventions involving trauma-informed Buddhist therapy.

*“An eye for an eye makes the whole world blind”—Mahatma Gandhi*

## Background

Acid attack, acid violence (AV), chemical assault (CA), or vitriolage is a cruel and pernicious form of violence, often leaving its targets with third-degree burns to their eyes, face, ears, and nose, while inhaled fumes can damage the viscera. Disfigurement is often permanent and survivors face isolation, stigma, and suicidal ideation (Azim & Zaman, 2023). In Asia, attacks on women’s faces are deliberate attempts at disfigurement (Siddika & Baruah, 2018, p. 158) to ruin her future romantic or marriage prospects. Potential mechanisms of death in fatal cases include septicemia and multiorgan failure (Byard et al., 2023).

Acid attacks are a global health issue but the phenomenon appears to have until recently been concentrated in South, Southeast, and East Asia; for example, in Afghanistan (Radio Free Afghanistan, 2012), Bangladesh (Das et al., 2015; Faga et al., 2000), Hong Kong (Young et al., 2002), India (Manizia & Mishra, 2022; Mittal et al., 2021; Rai & Pathak, 2019), Indonesia (Acid Attack on Students Again, 2013), Iran (Rahbari, 2022; Sabzi Khoshnami et al., 2017), Malaysia (Sim et al., 2011), Myanmar (Rigby, 2018; Thant Zin Oo, 2020), Nepal (Atreya et al., 2016; Lama et al., 2015), Pakistan (Bibi et al., 2021; Haroon et al., 2021), Singapore (Chin, 1980), Sri Lanka (Karunadasa et al., 2010), and Thailand (Asia News Monitor, 2012). A literature search identified 262 articles on acid attacks, 50 on vitriolage, 21 on AV, and 73 on acid burns (differing terms for the same thing) and found the studies evaluated barely dealt with the cultural drivers of acid attacks. Existing evidence regarding the underlying motives and triggers is fragmentary. For example, according to Mannan et al. (2007), the causes included financial and domestic disputes in Taiwan, rejected romantic advances by a woman to a man in Bangladesh, a woman attacking another woman on account of infidelity in Jamaica, and robbery and burglary in Uganda. According to Kornhaber et al.’s (2021) systematic review, the motives for attacks were varied and included the following: dowry-related, rejection or denial of sexual advances/marriage, property or monetary disputes, disputes between neighbors, and socially motivated reasons (family disputes, marital disputes, jealousy, infidelity, hate/enmity, domestic disputes, and revenge).

Along with Intimate Partner Violence (IPV) and other forms of physical aggression, acid attacks are generally considered as forming part of the spectrum of gender-based violence (GBV). My study arose from ongoing work with GBV and, given that the impetus for highlighting this important topic arose from gender-rights advocates and the fact that most of the published literature describes acid attacks as a form of GBV, there was a temptation to follow the same line here. However, just as a critical anthropology would caution against privileging culture, it seemed prudent not to jump to the conclusion that acid attacks are *necessarily* a form of GBV, and that, to resolve this question, it was essential to gain a better understanding of the cultural dynamics of acid attack in all its forms.

However, although the role of romantic jealousy is acknowledged, there has been no delineation of the range of categories of attack, the cultural context, or the local cultural and religious resources for prevention or intervention, nor has there been any systematic examination of drivers other than jealousy—for example, the role of envy. What is more, the privileging of western categories is problematic in itself, which leads to the fact that, although a number of studies mention ethnography, only a few of them (e.g., Díaz-Benítez [2021] seem to be based on ethnographic studies in any country).

This current study responds to this gap with a focus on Cambodia, one of the worst-affected countries in the world, with high levels of violence and impunity and with a social and cultural tendency to resolve conflict through disproportionate revenge.

Survivors of acid attacks in Cambodia, many of them blinded (Bendick, 2011; Hsin-Ju Lu et al., 2011) and as many as one-quarter young children (Gollogly et al., 2008), continue to suffer physical, social, psychological, and economic consequences (Licadho, 2003; Welsh, 2009), including severe social isolation and ostracism, and are sometimes driven to suicide (Gollogly et al., 2008). The Khmer media reports cases in gory detail but, because people think that many attacks arise from love triangle disputes, those attacked seldom receive much sympathy (Human Rights Watch, 2019).

It is claimed that Cambodia has seen an 80% reduction in attacks since the passing of the 2012 Acid Control Law but the reliability of these claims is questionable. There has been a significant decline in peer-reviewed publications on the issue, making objective assessment more difficult. Contrary to the self-congratulatory tone of the authorities, my personal experience is consistent with reports in social media, local Khmer-language news stories, and reports by NGOs involved in the care of survivors, all asserting that attacks continue to occur (Calcini, 2022; Eisenbruch, 2018b; Eisenbruch & Chhun, 2022).

The literature on Cambodia, as with the global literature, pays little attention to the cultural meaning of attacks and the broader lessons to be drawn about cultural responsiveness in GBV. I have been studying acid attacks in Cambodia since 1999, when the first cases occurred (Eisenbruch & Chhun, 2022) and, based on the 88 case studies I examined, have identified three taxa (Eisenbruch, 2025). The first taxon and most prevalent pattern (*n* = 56) was driven by romantic jealousy in the context of an explicit “love triangle” involving a married couple and rival. Commonly, the spouse, suspecting their partner of infidelity, hurled acid at them (*n* = 29). In most cases, the wife attacked her husband (*n* = 21), and the other cases involved the husband attacking his wife (*n* = 8). Almost as commonly, the wronged spouse attacked the rival (*n* = 24). In the second taxon, the attack was perpetrated as a form of IPV between the couple (*n* = 19). Usually, the husband abused his wife to the point that she attempted to flee the marriage and rejected his offers of reconciliation. In response, he hurled acid to maim her and prevent her from partnering with another (*n* = 16). In the final taxon, attacks were perpetrated in a wider social and political context of dysfunctional conflict resolution (*n* = 13), with fighting among members of a peer group, envy directed at another for their success in their business dealings, or during disputes over moneylending. These findings give rise to the focus of this study, aiming to explore the cultural context for acid attacks, leading to a more nuanced uncovering of the motives for their perpetration in each of the three contexts of violence identified above.

For Cambodia, little is known about the cultural drivers of acid attacks in particular, and GBV more generally (Eisenbruch, 2018a, 2019). In earlier work on the cultural epigenesis of violence against women in Cambodia, I have reported how perpetrators are driven by a series of sequential “cultural attractors” (Eisenbruch, 2018a, 2018b) or mutually reinforcing cultural mechanisms: (a) “blighted endowment,” or “bad building,” /sɑmnaaŋ mɨn lʔɑɑ/ (សំណាងមិនល្អ); (b) the legacy of deeds in a previous life, /kam/ (កម្ម), from Pāli, *kamma*; (c) astrological misfortune, /krʊəh/ (គ្រោះ), from Pāli, *grāha*; (d) astrological incompatibility in the partnership, /kuu kam/ (គូកម្ម ); (e) the “Triple Poison” of a perpetrator’s craving and greed, /lobha/ (លោភ:), from Pāli, *lobha*, anger and aversion, /toohsa?/ (ទោស:), from Pāli, *dosa*, and delusion, /mooha?/ (មោហៈ or មោហ៍), from Pāli, *moha*, also known as /avijjā/ (អវិជ្ជា), from Pāli *avidyā*, meaning unwisdom, misconception about reality; (f) “entering the road to ruin,” /ʔaʔbaayaʔmuk/ (អបាយមុខ), from Pāli, *apāyamukha*, meaning “facing the mouth of ruin”; (g) confusion and loss of judgment, /moohaʔ/ (មោហៈ or មោហ៍), from Pāli, *moha*; and (h) the “mind cloaked,” /moo baŋ/ (មោហ៍បាំង) and “mind shut to morality,” /moo bət/ (មោហ៍បិត), that is, moral blindness. Whether or not acid attacks are invariably a form of GBV, it makes sense to utilize these cultural attractors as a template to investigate the presumed motives for acid attacks in the present study.

A superficial and essentialist knowledge of local culture can lead to ethnocentric mistakes by outsiders. For example, a popular misinterpretation of karma in Buddhist societies (of which Cambodia is one) is that it traps the survivor in a cycle of “self-blame” for having brought their misfortune upon themselves through their conduct in a previous life, burdening them with a passive sense that they cannot escape their awful destiny. The findings reported here, however, echo the distinctly different interpretation of Buddhist scholarship on karmic theory: the perpetrator and survivor of GBV in general—as exemplified by acid attacks—have a mutual karmic entanglement with each other, and each makes sense of how they got caught up as a residue of their previous lives; armed with this knowledge, they can avoid further suffering in the future (Eisenbruch, 2018b, 2019). A similar level of cultural analysis is extended to some of the other “cultural attractors.”

To the best of my knowledge, this is the first ethnographic study of acid attacks in any country. This article delves deeply into the cultural construction of acid attacks, elucidates the cultural idioms of the complex emotions of jealousy and envy and how they are experienced, and illustrates how these cultural idioms are expressed in metaphorical language.

## Methods

The cross-cultural studies mentioned above, valuable as they are, have tended to rely on questionnaire and survey methodologies and seldom tap into the deeper cultural construction of emotions. The ethnographic methodology adopted in the present study allows for more in-depth exploration.

The meaning of romantic jealousy is embedded in the local belief systems, such as cause-and-effect. In Buddhist settings, karma, for example, is not simply about “blaming” a survivor for their conduct in a previous life or about leaving them with a passive sense that they cannot escape their awful destiny. I believe that a more nuanced understanding can allow us to see that perpetrators and survivors have a mutual karmic entanglement; each can make sense of how they got caught up in this life as a residue of their previous lives, and armed with this knowledge, understand how they can shrug off this legacy and reduce further suffering. A similar level of cultural analysis is extended to some other “cultural attractors.”

I write as a male, middle-class, medical anthropologist and transcultural psychiatrist based in Australia with an acquired understanding of Cambodian Buddhism and fluency in the Khmer language, but with sufficient cultural humility to appreciate the expression, “Khmer speech, foreign heart.” Since 1990, I have led a research program on traditional healers and Buddhist monks in the context of community mental health and GBV. The fieldwork encounters reported in this article were conducted between 1998 and 2022 by my Cambodian assistants (Mr CA and Ms CP) and me. Mr CA is a male native Cambodian. Through a long apprenticeship, I have trained him in the ethnographic method and have engaged in fieldwork with me in urban and rural areas for over 30 years. He has longstanding relations with many of the informants. Ms CP is a female native Cambodian with a background in Khmer linguistics who has worked with me for 12 years, in Australia and Cambodia. Other research assistants were recruited occasionally but not regularly. Wherever possible, women interviewed women, and men interviewed men.

This study is part of an ongoing ethnographic research project in Cambodia, focusing on GBV. Approval for various components of this study was obtained from the National Ethics Committee for Health Research in Cambodia and Monash University in Australia. In working with acid attack survivors, in addition to the usual ethical emphasis on participant safety and beneficence, we were mindful of the need to avoid unwittingly exploiting the survivors’ injuries and to be sensitive to the possibility of vicarious trauma, ethical issues noted by Pio and Singh (2016).

This study was conducted between 1998 and 2024. My research assistants and I followed an ethnographic path within the context of our ongoing engagement with communities, through which we developed close connections with particular members of the traditional healing sector and with villagers who were steeped in Indigenous knowledge and were willing to talk about it. Many of our connections have been forged over years of ongoing contact. In the focus on acid attacks, we included encounters with 169 informants from a variety of backgrounds: family members of those directly affected by acid attacks; perpetrators; Buddhist monks and ritual officiants, men steeped in cultural knowledge, experience, and wisdom about acid attacks; and female Buddhist devotees, some themselves survivors of family violence, with an intimate understanding of women’s experiences. The informants were recruited through snowball sampling in the following provinces: Battambang (3), Kampong Cham (21), Kampong Chhnang (3), Kampong Speu (9), Kampong Thom (13), Kampot (3), Kandal (20), Koh Kong (2), Kratie (3), Phnom Penh (34), Pailin (1), Prey Veng (8), Pursat (15), Takeo (18), Tbong Khmum (10), Banteay Meanchey (2), and Svay Rieng (4). The average age of those attacked was 40 years.

There were 88 cases of acid attacks. Some incidents involved multiple perpetrators and survivors (or fatalities), giving a total of 102 perpetrators and 97 targets. The socio-economic backgrounds of perpetrators and those attacked are shown in Table 1.

**Table 1.** Socio-Economic Backgrounds of Perpetrators and Targets.

Socio-economic background	Perpetrators	Targets	Total
Business or trading (hairdresser, food retailer, cosmetics, moneylender)	19	24	43
Unskilled worker (laborer, hairdressing, casino, food stall holder, plantation worker, tuk-tuk driver)	21	18	39
Garment worker	7	14	21
Housewife	15	4	19
Farmer (rice, fish, vegetable, orchard)	7	9	16
Services (administration, soldier, waitress, government official, karaoke)	2	13	15
Gangsters	11	0	11
Child	0	6	6
High status—military or political power or wealth (or wife of)	4	1	5
Buddhist clergy	1	2	3
Unknown	16	7	23

All participants gave at least verbal consent, and they read (or were read) a Plain Language Statement about the project. Everyone agreed to participate, and no one dropped out at any point. The researchers were not involved in the patient care of those affected. The encounters varied from single meetings lasting 1 hr to more intensive and repeated ones, and interviews were conducted at individuals’ homes, with as much privacy as possible. Most of our encounters were with survivors and their families, and were respectful and mindful of the stigma that many of the survivors faced; others were with perpetrators, and with the monks and traditional healers survivors had consulted.

The interviews with the survivors and their immediate relatives were open-ended, and we explored their lived experience and understanding of the reasons for the attacks. The following are examples of the questions posed by the interviewers: (a) Were there signals, once the survivor got to know the perpetrator, such as the existence of a mole on the genitalia? (b) Had the perpetrator shown evidence of being vicious as a child as a forerunner to perpetrating an attack in adulthood? (c) Had the attack taken place during a period when the survivor, based on their astrological state, was more vulnerable to mishap (krʊəh)? (d) Was there a perceived mismatch between husband and wife based on their respective zodiac houses and which would predict the inevitability of a rupture in the relationship? (e) Did the perpetrator exhibit an excess of the three Unwholesome Roots (akusala)? (f) Had the perpetrator entered the “road of ruin,” exhibited by the triad of gambling, drinking, and womanizing?, and (g) Had the perpetrator, driven out of their mind by anger, jealousy, or envy by, for example, becoming suspicious that they were being cuckolded, been overcome by emotions of grief, anger, and revenge? We paid attention to their cultural registers, such as concepts of karma and the use of popular Khmer cultural references. We gave them time to work through these feelings, minimizing the chance of traumatizing them. Many were relieved that after the passage of time, their personal stories mattered and were sufficiently important to be heard. Even the discussions with perpetrators were carried out with sensitivity to their feelings of hurt, sometimes of actual physical abuse, which had led to the attacks.

As far as possible, we developed an ongoing connection with the survivors and their families, especially where specialist organizations had withdrawn their services. We followed the trajectories of some survivors, especially when they had exhausted all possible interventions to surgically resolve their physical scars, in their search for relief from the local Buddhist temple and from traditional healers. We followed up with these monks to explore their perspective on the causes of the attack, and also on the implications of their approach. For example, if the monk emphasized to a survivor, as well as to us, the role of karmic destiny, we would note how he conveyed to the survivor the importance of “letting go” any smoldering feelings of revenge, and we would observe how this advice helped relieve the patient’s mental suffering. Assistance was offered, for example, by taking survivors to health and support services. All encounters took place in Khmer.

The author, aided by the research assistants, relied on grounded theory and conducted a modified thematic analysis to seek the insider views of people within the culture and a narrative inquiry of the storied lives of case studies (Bhattacharya, 2017; Levitt et al., 2018; Pham et al., 2021). The starting point was “theoretical sensitivity” (Thistoll et al., 2016), acquired through years of fieldwork experience with child sexual abuse in Cambodia. The process of data collection, analysis, and discussion was conducted in Khmer, after which the relevant materials were translated into English. In this ethnographic work, the process was iterative rather than linear, evolving through the inquiry, with movement between data collection and analysis (Levitt et al., 2018). We analyzed the cultural idioms of acid attacks using qualitative data techniques that ensured fidelity and minimized observer cultural bias, and we promoted methodological integrity using supplemental checks in which transcripts were iteratively discussed with some of the informants. We also analyzed the linguistic structures of romantic jealousy, envy, and revenge. In a process of iterative discovery, we followed up with informants, in some cases for as long as 15 or more years after the initial encounter, and we listened to the evolution and reshaping of their own narrative interpretations of the meaning of the acid attack. Participants were de-identified, and the names used in this article are pseudonyms.

By treating the Khmer terms as local concepts and idioms and taking them as a starting point, we avoided the “category fallacy,” defined by Kleinman (1987, p. 452) as “the reification of a nosological category developed for a particular cultural group that is then applied to members of another culture for whom it lacks coherence and its validity,” and D. E.Hinton et al. (2013) speak of this error as “category truncation.” Rather than transliterating, Khmer terms are spelled using Huffman et al.’s (1970) adaptation of the International Phonetic Alphabet transcription to help non-speakers pronounce the terms easily and consistently. In each case, the word is shown in parentheses in KhmerOS font. Words in Pāli are written in italics. Pseudonyms are written in the sequence of family name followed by personal name.

## Cultural Framing of Acid Attacks

### Drivers

#### Karma

The popular belief is that acid attacks are the result of a person’s intentional karmic action /kammaʔ pʰɑl/ (កម្មផល) in this or a previous life and that the perpetrator and survivor were already intertwined through their previous lives. Ty Vy had built up a successful business because of her people skills. Her success aroused the suspicion of the wife of one of her customers. Believing Vy had beguiled her husband, she decided to use acid to destroy her supposed rival. One night, Vy dreamt that her mother’s spirit had come to watch over her in her sleep. Moments later, the woman threw acid on Vy, waking her up. As she woke, Vy found that, mysteriously, her arm had moved automatically to her face, deflecting the acid and causing some to splash back onto her assailant. Vy understood that her mother’s spirit had controlled her arm to move this way. She wondered whether the attack was a consequence of the karmic debt she had accumulated to the assailant in their respective previous lives. If this were indeed so, she prayed that her physical and mental scars would be paid in full. However, if this were not the case, she prayed that the perpetrator should pay for the consequences of her action by “serving her” for their next life and the one after that. Vy discharged her outrage and called for “justice” without drawing herself into infinite conflict. Unlike her perpetrator, she overcame her natural tendency to allow the desire for vengeance to consume her.

#### Endowment

I have described elsewhere a traditional Cambodian belief that a man endowed from birth with one or more penile moles possesses a sort of “natural” love charm that has the magical power to “lasso” women into toxic relationships and coercive control, creating fateful love triangles and marital misery, which can ultimately lead to an acid attack by a jealous partner (Eisenbruch, 2024). There were at least ten cases of acid attacks perpetrated in the context of triangular love in which a penile mole was said to have been instrumental.

Usually, a man with a penile mole “lassos” a woman and exploits the situation to start a relationship and, when his wife discovers it, she throws acid at her rival’s face. In another pattern, the wronged wife throws acid at her husband. Soon after Sry Sophoan started to cohabit with her common-law husband San Soriya, she discovered that he had no less than seven penile moles. She said that these moles were powerful magnets that enabled him to “lasso” at least 25 women in a series of relationships. A tuk-tuk driver who would often journey away from home, people said this kind of man would pick “spare wheels” or, using the colonial French, “*roues de secours*.” Sophoan said that not only did the penile mole lasso women, but it also made him sex crazy. She said that sometimes he brought two girls to sleep in the same bed as her, together under the mosquito net. What could she do, she said, she would try to sleep on the edge and let him do what he wished. He shamelessly asked her to allow him to have another wife, as his “spare wheel.” She could not compete with Soriya’s moles and, in the end, all she could do was to throw acid at him.

#### Character

There is a tendency for women to blame the bad character of rivals. Some women were seen as gullible. A man whose daughter had been fatally attacked with acid by a wronged wife after she had an affair with a married man said that his inexperienced daughter had naïvely fallen prey to the convincing words of the predatory man; “her heart dropped straight down,” /cət sloy/ (ចិត្តស្លុយ), a metaphorical expression as if her reasoning and emotions were like a traditional Khmer skirt in which the fabric has a straight drop to the ground.

Predatory women characterized as seizing married men were depicted using idiomatic expressions such as “a dog lassoing men,” /srǝy cvak proh/ (ស្រីឆ្វាក់ប្រុស). Some men who had survived the attack at the hands of their wives said that, far from being “the virtuous woman,” /srəy krup leakkʰaʔ/ (ស្រីគ្រប់លក្ខណ៍), their wives were “flawed” and had “lost their perfection” /srəy kʰaat leakkʰaʔ/ (ស្រីខាតលក្ខណ៍). Some monks said these patterns were found in the Sujāta Jātaka, (7:59; IV 91–94), known in Khmer as /soʔcietaa ciedɑk/ (សុជាតាជាតក). Some informants quoted the first three of the seven types of wives listed in it: the destructive wife, fond of other men and contemptuous of her husband; the thievish-wife, who is dishonest with her husband by squandering the family wealth; and the tyrant-wife, who is shrewish and domineering.

In another metaphorical image, Vy, mentioned earlier, thought that her assailant, who was from the Thai border, was a woman like all women on the border, with “the heart of a tiracchāna” (តិរច្ឆាន)—“a horizontal-walking beast inhabiting the netherworld.” From this, she formed the view that village women were collectively worried that their husbands were playing around with other women and that they had incited one another by picking a woman, in this case herself, to gossip about as the culprit who was stealing their husbands. Thus, she naturally became a target. By disfiguring her, her assailant had made her the scapegoat for the other women, too.

The character of incorrigible married men who incessantly drew women into poisonous relationships with them, only to discard them after the women had become damaged (sometimes by acid attack), was typified by the deceptive manner in which they had drawn these women into a toxic relationship for which they paid by being attacked with acid. In this idiomatic expression, which is also a play on words, this sort of man is literally, “sneaky, sly” + “broken, damaged” + “woman,” /kʰəl + kʰooc + srǝy/ (ខិល + ខូច + ស្រី). The implied meaning is that the man will be broken (maybe by acid attack) and that he, too, will damage other women in the process who, in turn, will become “broken women,” /srǝy kʰooc/ (ស្រីខូច), the same word as is used for a prostitute. The men who throw acid at the mothers of their children are similarly designated. One would think that an acid hurler might think twice about exposing infants and young children (sometimes their own!) to their attack, but I never heard of a single perpetrator who hesitated on account of the collateral damage they were likely to inflict on the child. People describe the lowest of the low as having “the heart of a tiracchāna,” /cət tiracchāna/ (ចិត្តតិរច្ឆាន). This is a term drawn from Buddhist cosmology, and refers to a beast that, unlike a human, moves horizontally and has no aspiration to move upward in morality, as I have described elsewhere in the cases of men who abuse their children (Eisenbruch, 2019).

#### The Triple Poison

The more knowledgeable informants said that men who were targeted for attack, whether because of getting into a love triangle or simply for beating their wives, had an excess of one or more of the Buddhist “triple poisons.” The informants highlighted an excess of two emotions, anger and possessive jealousy, as fueling attacks. Phan, a married man who had been driven crazy for a woman who cast a love charm on him to exploit him, said that it had made him fall into the pit of the Buddhist Triple Poison, or the three fires: greed, hatred, and delusion.

In triangular love, the person attacking their spouse for their presumed cuckoldry is consumed by “the fire of possessive jealousy,” which, in turn, is one of the Triple Poisons, the poison of sexual desire, lobha (លោភៈ). Their “slow fire of revenge” is connected to another Triple Poison, that of destructive anger, dosa (ទោសៈ). Informants described them as literally “hot of heart-mind,” /cət kdav/ ().

#### Astrological Risk, *graha*

A person can face an astrological risk of being attacked. Ky Troeung and his wife Tou Sim had been in a physically abusive relationship. He repeatedly ran off with other women whenever she was pregnant, and they had separated several times. Troeung would always come back, morbidly jealous, and he had nearly killed her a few times. For example, he ran after her with a meat cleaver after accusing her of being unfaithful and smashed her phone as he suspected she used it to communicate with this unknown man. Even after they got a divorce, Troeung continued to plague her.

Sim consulted an astrological fortune teller (kruu Saem) to find out how to get rid of him once and for all. Using his horoscope manuals, the healer made a series of calculations. Sim was born in the Year of the Goat and was then 42 years old. He divided 42 by 7, and the result, 6, was a pure integer, with zero remainder. In a second calculation, he multiplied 42 by 3 to arrive at 126 and then divided that by the 9 Navagraha, the 9 heavenly planets, resulting in the pure integer 14. Again, there was zero remainder. With this doubling, the fortune teller diagnosed an intensification of Sim’s “heavy *graha*.” Quoting the saying, “If, in an effort to escape your *graha*, you enter a remote forest, there are thorny plants. If you enter a crowded village market, there are policemen waiting to arrest you,” /cool prey bɑnlaa, cool psaa poolih/ (ចូលព្រៃបន្លា ចូលផ្សារប៉ូលីស), he said it was hard for Sim to escape. What was more, Sim could not afford the cost of performing the ritual to “disentangle herself and the astrological misfortune of Number 0 from one another,” /rumdɑh krʊəh trəv comleek soon/ (រំដោះគ្រោះត្រូវចំលេខសូន្យ). Sure enough, late one night when his last effort at seeking reconciliation had been rebuffed, Treoung attacked Sim and their children with acid.

#### Astrological Incompatibility

**Figure 1.**
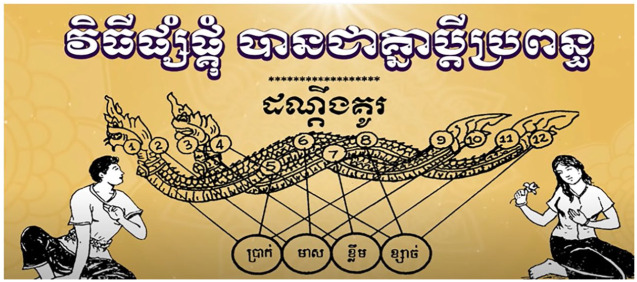
Part of material used by healers and available at stalls in markets titled “Sɑmpʊəŋ Of The Nāga” (astrological goodness-of-fit in the relationship between a prospective couple, based on the astrological Nāga system).

Acid attacks, love triangles, and possessive romantic jealousy were written in the stars of a misaligned couple. In 2018, Song Vanna, a construction worker aged 30 years, and Yeang Sophany, aged 21 years, had fallen in love and were living together. The two sets of parents had their worries from the start. Sophany’s parents were disturbed that Vanna was habitually drunk and would beat up their daughter, and it was reputed that he would hook up with many women on his road trips, so they broke off the engagement and looked for a better match for their daughter. At first, Vanna accepted this—until he discovered that Sophany had a new suitor. On the day of the public announcement of the engagement, overcome by jealousy and convinced that Sophany had two-timed him during their relationship, Vanna rode his motorbike to her house and, when Sophany emerged, threw a bottle of acid on her, and swallowed the rest. The family made sense of this double tragedy. Sophany, born in the Year of the Tiger, and Vanna, the Year of the Rooster, should never have been together. Vanna’s mother had consulted a fortune-teller, /kruu tiey/ (គ្រូទាយ) about the match, and he consulted his “Sɑmpʊəŋ of the Nāga’ Manual” (Figure 1).

The diagram shows a pair of intertwined Nāga serpents, with the heads to the left. The fortune-teller counted each of the 12 zodiacal years. For Vanna, he started at “number 1” at the head of the first Nāga and, given that he was born in the Year of the Rooster, the fortune-teller landed him on number 10, at the first Nāga’s tail. Then, the fortune-teller counted the years for Sophany, who was born in the Year of the Tiger. He counted from the tail forwards of the second Nāga and landed on “number 3” near its head. The prediction was obvious: the wife-to-be, sitting at the head of one Nāga while her husband-to-be sat at the tail of another meant that the husband would easily be dominated by his wife. The orientational metaphor is a reversal of the traditional Khmer expectation that a husband should be at the head.

Vanna’s mother had urged him to give up Sophany, but he told her that he would gamble on following his heart—he used the Khmer term, which literally translates to when the dealer in the card game sweeps in the money from the losers, /sii sɑɑŋ/ (ស៊ីសង). Vanna’s love was blind; he was like a barnyard fowl caught in the jaws of a tiger, while Sophany was ever hungry for bigger prey, even an elephant. As his uncle and mother told us later, the fowl knows to keep out of the tiger’s reach by flapping its wings to fly to safety but, tragically, Vanna, unlike other more prudent fowl, remained on the ground in harm’s way. Vanna’s fate was obvious to his mother and uncle. Sophany’s cuckoldry was in the stars, and she cracked a whip and “flicked him off”—she used the Khmer term /rɔloah/ (រលាស់).

#### Moral Blindness

Moral blindness was the final step in the cascade of cultural attractors. Commonly, the perpetrator would say that heavy drinking had made them this way, certainly during the moments leading up to the attack. Morn Vitheavy, 35, had kicked out her husband Yeang Yoeun, 40, for his heavy drinking when he would beat her up and she would refuse sex, so he jumped to the conclusion that she had other men and, morbidly jealous, he decided to destroy her face. In a drunken frenzy, he smashed the bottle of acid on Vitheavy’s face to teach her a lesson. Later, Yoeun said that because of the alcohol, his mind had become /moo bət moo baŋ/, that is, the door to his mind had been closed, /bət/ (បិទ), as if the mind has an umbrella over it, from the word /baŋ/ (*បាំង*), which means “to cover.” His mind could think of nothing other than to hurl the acid, with no regard for the consequences, even if he ended up in prison, and no-one could hold him back. Subsequently remorseful, the instant that his mind woke up, he knew at once that he had done wrong, saying, “a word came from my mouth, it was wrong in a minute, it was wrong at that time, and so it was wrong for the rest of my life.”

In real life, the various cultural attractors and socio-economic circumstances synergize. Hy Cheng and Leng Reaksmy, 7 years his senior, were adolescent sweethearts, but Reaksmy’s father had sized up Cheng’s family background as a bad one, so he did not consent to the match. True to form, Cheng, inflamed by Reaksmy’s father’s action, threatened, “One day, I will buy gasoline and burn down your house! You wait and see! You will die in your house.” Decades later, it became apparent that this was no idle threat. Reaksmy and Cheng had gone on to marry and divorce other partners and they found one another once more and began living together. Over a period of 10 years, however, Cheng descended into drinking and gambling and severely assaulted Reaksmy. She reported the incidents to the police, but all they did, as was customary in Cambodia at the time, was to urge the couple to reconcile. Finally, Cheng abandoned Reaksmy and their children to be with his other “wife.” It was 2020, and despite the COVID-19 ban on travel, Cheng kept returning to Reaksmy to try to have sex with her, but she refused. For one thing, she might contract COVID, or even AIDS. Rejected and infuriated, Cheng reminded her how her father had interfered when they had been teenagers, and if he discovered that her father was the reason for her rejecting him now, he would incinerate him. Reaksmy was about to report the threat to the police but too late. Cheng, redirecting his rage, hurled acid at Reaksmy, punishing her and her father in the bargain.

During her recovery, Reaksmy reflected on why her life had been so. Consistent with the popular belief among those of her rural background, she told us that she had been born with a tear mole, literally “mole that is the scaffolding for tears,” /prɑcruy rɔɔŋ tɨk pnɛɛk/ (ប្រជ្រុយរងទឹកភ្នែក), under her left eye (of course, all that was no longer apparent as her face had been destroyed), and throughout the years of abuse, had long suspected that she was marked as a “a woman of misfortune,” /srǝy ʔaʔpʰoap/ (ស្រីអភ័ព្វ), a suspicion confirmed by the acid attack. While suffering so, the memories of Cheng’s threat to her father flooded back. How ironic, she must have thought, that the threat of her father’s house being incinerated had come true with the incineration of her own face.

Cheng fled and went underground until he caught COVID-19 and was sent to the COVID-19 hospital, where he was immediately spotted, arrested, and sentenced to prison. His incarceration provided cold comfort to Reaksmey, however, because she was terrified that, upon his release from prison, he would be back to wreak more vengeance upon her family.

### The Language of Jealousy and Envy

The two main complex emotions fueling acid attacks are romantic jealousy and envy, and these emotions are expressed through primary emotions: anger, fear, hopelessness, and despair. The language of these emotions—the linguistic expressions at lexical, semantic, pragmatic, and discourse levels—reveals how the Cambodian informants construe the cultural architecture of acid attack and, beyond that, the dynamics of intimate relationships, violence, and their theory of mind.

Anger is a central feeling in jealousy, in which the person experiences anger towards their rival or the loved one whom they have lost, and in envy, in which the anger is directed at the person who has what they desire. The anger is depicted metaphorically by the informants as fire and heat, which matches the flavor of news media reporting on cases of acid attack caused by “the fire of jealousy” /pləəŋ prɑcan/ (ភ្លើងប្រចណ្ឌ) which burns and spreads.

The acid attack perpetrated by the man spurned by his former wife (“if I can’t have you, nobody can”), and filled with possessive jealousy and rage (“you cuckolded me with another”) involves an intensification of hot rage—love turned to hate—and such heat can be rapidly stoked by inflammatory language.

The target of an envious attack is seen as a threat to be disposed of. For example, a talented, attractive, and successful newcomer who enters a community is a threat to the inhabitants, and is perceived as a rival and a threat, a “wild forest hen”/srǝy moan prey/ (ស្រីមាន់ព្រៃ), whether in business or love.

### The Love Triangle and Love Magic

The first taxon is the love triangle (Table 2).

**Table 2.** Romantic Jealousy in an Explicit or Suspected Love Triangle.

Group	Perpetrator	Target	Timing
Group 1: Jealous wife/husband, suspecting their partner of infidelity, hurled acid at them (*n* = 29)			
Wife on husband (*n* = 21)			Still married (*n* = 17)After marriage ended (*n* = 4)
		
Husband hurled acid at his wife (*n* = 8)			After marriage ended
			Still married
Group 2: Explicit love triangle and wronged spouse attacked their rival (*n* = 24)			
Wife attacked female rival (*n* = 22)		Female rival	Still in relationship or married (*n* = 14)
		Female rival	Estranged (*n* = 6)
		Female rival who also had her own husband	After the husband abandoned his wife for a rival (*n* = 1)
		Female rival from an earlier “bigamous” relationship	Still in a relationship (*n* = 1)
Husband on rival mistress (*n* = 2)	Husband (*n* = 1)	Mistress (who had chased him out)	Still in a relationship with the principal partner
	Husband (*n* = 1)	Mistress (sought a new relationship beyond him)	Still in a relationship with both his wife and lover
Group 3: Explicit love triangle, and rival attacked spouse (*n* = 2)			
	Female rival (*n* = 1)	“Wife”	Still in a relationship with the principal partner
	Female rival (*n* = 1)	Husband and a subsequent partner	Estranged
Group 4: Husband’s agent sets out to attack the suspected male rival (*n* = 1)			
	Presumed husband’s agent (*n* = 1)	Innocent monk, mistaken identity	Unknown

**Figure 2.**
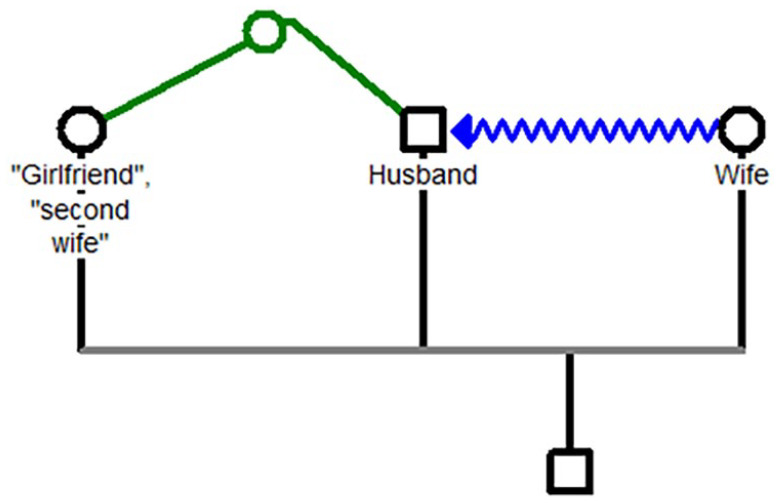
Taxon 1. The green line represents the attraction between the husband and the other woman. The blue line represents the reaction of the wife, specifically, hurling acid at the husband.

**Figure 3.**
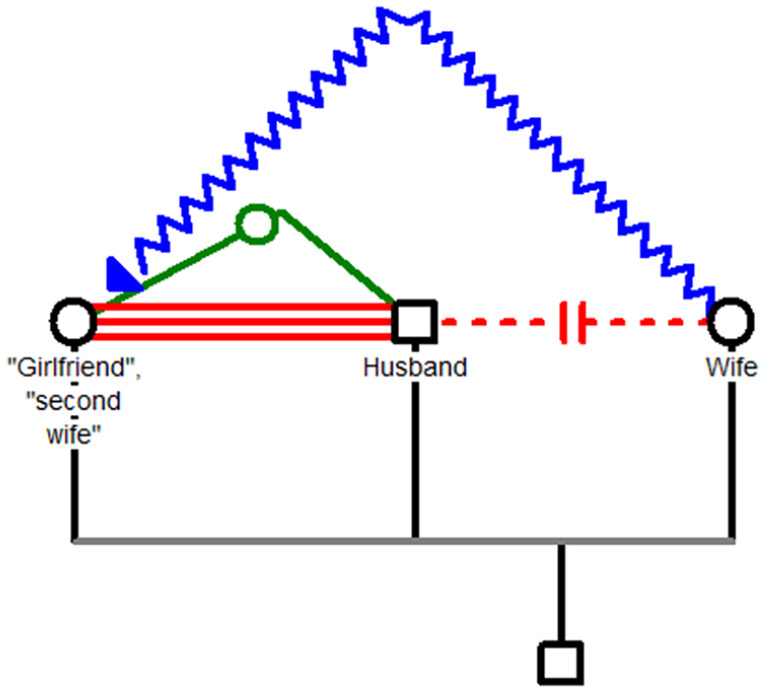
Taxon 1. The red line represents love charms cast by the rival to attract the husband to her and repel him from his wife. The green line represents attraction between the husband and the rival. The blue line represents the reaction of the wife—hurling acid at the rival.

The two main patterns in the first taxon are illustrated in Figures 2 and 3.

A common belief among wives wronged by their husbands was that the rival had cast a love charm to ensnare their husbands. To return to the case of Sry Sophoan and her common-law husband San Soriya, in her desperate efforts to bring him back, Sophoan had consulted a traditional healer who prescribed and gave her two sorts of love charms. Sophoan was sure that her rival had interfered with the marriage by casting a “love charm to destroy and separate” /snae bɑmbaek/ (ស្នេហ៍បំបែក) Sophoan and Soriya. In response, Sophoan ran to a traditional healer to cast the same type of love charm to destroy the attraction between Soriya and her rival, his current girlfriend.

In a parallel process, in which Sophoan wanted to pull Soriya’s heartstrings back to her and their children, she also cast a love charm called “love charm to put back together,” /snae bɑŋruəp bɑŋruəm/ (ស្នេហ៍បង្រួបបង្រួម) which had two components: the first, a “love charm to make the heart tender,” /snae bɑntʊən cət/ (ស្នេហ៍បន្ទន់ចិត្ត), and the second, more explicitly sexual, to revitalize the sexual energy between herself and her much younger husband, literally a “love charm to arouse and entwine,” /snae prɑteʔpoat/ (ស្នេហ៍ប្រតិព័ទ្ធ), a formal euphemism for having sex. With this, Soriya and the children physically dragged him back home time and again. Each time that her efforts eventually fell apart, Sophoan drew the conclusion that whoever was her rival at the time had cast love charms to counteract hers. Finally, Sophoan resorted to throwing acid at her husband.

The love triangle is not simply a question of two people, one or more of them being married, voluntarily falling in love. In Cambodian culture, it is depicted as one member of the triangle having been “lassoed” into becoming “lovestruck” by a love charm (Table 3).

**Table 3.** Selection of Cases in Which Love Triangle Was Attributed to a Love Charm.

Group	Perpetrator	Target	Timing	Love Charm
Group 1: Jealous spouse suspecting their partner of infidelity, hurled acid at them (*n* = 30)				
	Wife	Husband	Still married	Yes (by rival, and by rival and wife)
	Husband	Mistress (who had sought a new relationship beyond him)	Still in a relationship with both his wife and lover	The man had bewitched the woman who wanted to leave him to retain her as his mistress,
Group 2: Explicit love triangle and wronged spouse attacks their rival (*n* = 23)				
Wife attacks female rival	Wife	Female rival who also had her own husband	After the husband abandoned his wife for a rival	Man had penile moles, or the woman had genital moles and “lassoed” the opposite-sex partner
		Female rival	Still in relationship with principal partner	The rival had bewitched the husband
		Female rival from earlier “bigamous” relationship	Still in relationship	The “wife” sought to terminate the ongoing relationship between her husband and his former partner, and later, after the acid attack, the rival used a love charm to keep the bond between herself and her children with their father
		Female rival	Estranged	The man had bewitched his target woman who succumbed to his charms, and then his wife attacked her
		Female rival	Husband estranged from his “wife” while in the new relationship with his mistress (First triangle)	Two subsequent love triangles involving the acid attack survivor who used love charms to (1) ensnare a subsequent man, and (2) induce that man and his wife to break up

In February 2019, Ky Phea, aged 43 years, tried to behead and then hurled acid at garment worker Sean Chany, aged 23 years, killing her. He fled the country but was eventually arrested and sentenced to 14 years’ imprisonment. Chany’s cousin, Sineth, told us the story. Phea was a wealthy duck trader and holder of multiple passports; his wholesale empire stretched across the Mekong countries. A married man with a family, he was in the habit of lassoing women. A virtuous and frugal garment worker, Chany was not the sort of woman who would pursue Phea for his money. Even though Phea had abducted and raped her, Chany remained under his thrall. Phea, a business tycoon, presented himself as if he were a traditional healer, his house adorned with a tiered altar to his healing masters. People said he had drilled out his canine tooth and inserted a “magical love charm,” which imparted irresistible sweetness to the words that came out of his mouth. This was Chany’s weak point, said Sineth. She was ensnared by his dental magic, even after he raped her. It was rumored that Phea had a penile mole, which could magically “lasso” a series of women who would fall under his spell, even to the point of his exerting coercive control over them.

Four years went by, with no prospect of Phea leaving his wife for her, and Chany negotiated with Phea to let her go. Soon enough, Chany found a single man who wanted to marry her. When Phea discovered this budding romance, hotly jealous, he tried to behead her with a machete. When she put up her hands to shield herself, he pinned her hand and chopped at her left wrist repeatedly, where the ring was still on her ring finger, and shouted that he would not stop until he had severed her wrist. He hacked off her left hand and, when she fled, pursued her and hurled acid in her face.

### Omens

Sometimes, a simple object was taken to be an omen of attack. When Phea asked to meet with her, Chany decided to pawn the gold ring he had given her. However, it was stuck firmly on her finger. Phea then attacked her, and when her family arrived, they found her lying in a pool of blood, her hand on the ground where it had been chopped off, the ring still sitting tightly on her finger. They believed that Phea’s gift was an omen of the acid attack and murder.

A dream was also commonly taken as an omen. Meng Sopha and her husband had a flourishing cosmetics business. Another family set up a rival business next door, provoking quarrels and violence between the families. One night, Sopha had a nightmare in which the corpse of a murdered woman was delivered to her house and hoisted in a seated position high up, near the roof, with fresh blood dripping non-stop onto the house. The nightmare continued with Sopha, and her husband accused as the killers. When she woke up, Sopha feared that this dream was an omen of imminent disaster. Within a week, the rivals had thrown acid at Sopha and her daughter, and Sopha became suicidal. It was as if the dripping blood represented the acid falling on her and the corpse represented herself as she thought to hang herself from the high roof beam.

Most omens are more complex than these two examples, as acid attacks are usually part of a tapestry of “perils,” /cɑŋray/ (ចង្រៃ) affecting a family. A few weeks before his wife, Chheang Chhaya, was due to give birth to their child, Chay Om had started an affair with Khat Thida, and within days of the birth, Thida threw acid at Chhaya and her newborn.

#### First Omen: Anomalous Pillar, Bark Growing Within Heartwood, Signifying the Blighted Foundation of the House

The “peril” started years before the acid attack, when Om had been helping construct his mother’s house. He noticed bark growing into a cavity in the heartwood of the central pillar, an omen that this was a “house of peril,” /pteah cɑŋray/ (ផ្ទះចង្រៃ).

#### Blighted House, Leading to “Woman Who Brings Peril.”

The /cɑŋray/ continued, as Om, like many other men, slid into *apāyamuk*, drinking and womanizing. Om, an ox carter, was said to be the kind of man who “lassoed women.” Of all the “loose women” he could have met, however, Om’s /cɑŋray/ befell him when he met Thida, a street-seller who sold alcohol, whom Chhaya’s family and Om depicted as a “woman who brings peril,” /srəy cɑŋray/ (ស្រីចង្រៃ), a woman who hooked up with married men.

#### Second Omen: “Pregnancy Dream” of Two Children Fighting

At the beginning of her pregnancy, Chhaya had a dream of two children fighting, with a “broken face, broken mouth.” Chhaya interpreted the dream as an omen of the family fighting and later concluded that it had portended that her own and her baby’s faces would be burnt by acid.

#### “Woman Who Brings Peril” Leading to Disastrous Triangular Love

Om started an affair with Thida, who demanded him all to herself. According to Chhaya and other women, the evil Thida cast a love charm on Om to inflame his heart and “lasso him” to her. Chhaya believed this affair came about because of her husband’s bad character, a magical “lassoer of women,” /cvak srəy/ (ឆ្វាក់ស្រី) and his lover, a “lassoer of men,” /cvak proh/ (ឆ្វាក់ប្រុស), and that they had lassoed one another. As her mother put it, “they ran to one another.” Chhaya and her mother used the metaphoric expression, “If the pole with the hook that is utilized to pull fruit off the Malabar tree reaches up to it, the fruit will drop,” /tumpʊək vie tɨv təəp pnɨv vie mɔɔk/. Om initiated the connection, and Thida was hooked, they were both at fault. Chhaya believed her husband and his lover were driven by the Buddhist Triple Poison; for example, too much *lobha*, or lust, each pursuing multiple partners. She said that Thida had a “thick face,” meaning that she was not ashamed—an ironic image, given what Thida had done to her face. She added that her husband had entered *apāyamuk*, characterized by his alcoholism, womanizing, and gambling.

#### “Woman Who Brings Peril” Seeks to Murder Wife

Thida discovered that Om was about to become a father. With barely a week left before the birth, Chhaya had been staying at her mother-in-law’s house where the baby was to be born. Thida, armed with an axe, turned up at the family home intending to hack Chhaya to death. However, Chhaya held her ground and told her to get out. As Thida had no money to pay the fare home, Chhaya borrowed money from the neighbors to cover the amount and sent Thida on her way.

#### Third Omen: Snake Slithering from the Back to the Front of the House

As Chhaya turned to go back to the house, she saw a snake enter from the back and exit via the front, a bad omen calling for urgent action. The snake had entered their house to warn the family of impending disaster at the hands of the “woman who brings peril.” The peril had to be averted, and quickly, by inviting a monk to perform a ritual to “sweep out the danger,” “banish the headless ghost from the house,” /bɑndəɲ kɑmraol cəɲ/ (បណ្ដេញកំរោលចេញ), or “banish the peril” /bɑndəɲ cɑŋray/ (បណ្ដេញចង្រៃ). In this substitution ritual, the peril would have been symbolically transferred to the snake, carrier of the bad omen, to take it away from the village as it slithered to its home in the dangerous forest. The family failed to perform this ritual. Chhaya’s father believed that she, being the only member of the family who had seen the snake, should have warned the others. Perhaps she had too much on her mind, what with coping, during the pregnancy, with her husband’s affair and the traumatic encounter with her rival, and in any case the family could barely afford the 20,000 riels the ritual was said to cost. The baby’s birth was also imminent. This failure is made even more puzzling by what the traditional birth attendant (TBA) told us 10 years later in a follow-up interview. She said that even poor families like this one could have afforded to avert the bad omen signaled by the snake. All it would have taken was a simple process, for example, wrapping a bundle of old trousers, a sarong, or other rags and hurling them at the snake as it slithered back into the forest while uttering a simple stanza in Khmer, and the “peril” /cɑŋray/ would have departed with it.

#### “Woman Who Brings Peril” Throws Acid at Wife

Chhaya was apprehensive about the impending childbirth as her mother had died after giving birth. The death was attributed to the /priey krɑlaa pləəŋ/ (ព្រាយក្រឡាភ្លើង), lethal ghost-spirits who attack postpartum women for their malodorous lochia of blood, mucus, and uterine tissue. Chhaya’s family was sensitive to these ghost-spirits, which had killed her mother during childbirth. Therefore, the TBA carefully instructed Chhaya to protect her from the same fate. Om, her wayward husband, acknowledged his serious responsibilities at this time and came home. Their baby girl was born. Om, on the alert, did his husbandly duty to guard against the /priey krɑlaa pləəŋ/.

Impatiently, Thida awaited Om’s return. It had been 3 days since the birth, but he still had not come. Her vengeance was ignited. She purchased a liter of acid from a gold merchant for 25 cents. In the dead of night, Thida arrived and found Chhaya lying on a bed over a cauldron of steaming water, the “bed of fire,” as was the custom for women have just given birth, with Om dozing nearby to protect her from attack. Thida threw the acid on Chhaya and her baby. Awakened by his wife’s blood-curdling screams, Om instantly thought his wife had been attacked by the ghost-spirits and clutched her to his chest, and thus also absorbed some of the acid. Then he realized that it was Thida’s doing, so he dropped his wife and ran off in pursuit. Chhaya was gravely injured, and one breast had to be amputated. She and her baby were sent to Takeo Provincial Hospital, starting an arduous series of hospitalizations. Thida was sentenced to 8 years and gave birth in prison, perhaps to Om’s baby. Once the love charm wore off, Om felt fury at Thida for what she had done and said that if he were to visit her in prison, he would murder her. His love had turned to hatred.

#### Fourth Omen: The “Ghost-Dove” Perched on the House, an Omen of Double Death

The peril spilled over to include others. One night, while Chhaya was away receiving further surgery for her burns, a neighbor observed a nocturnal “ghost turtle dove,” /lɔlɔɔk kmaoc/ (លលកខ្មោច) perched on the roof of the Chay house, and the next day, the neighbor was dead. Chhaya, too, made a connection, remembering that the day after she had seen the snake, her mother-in-law fell ill with a fatal illness.

#### The Marriage Further Imperiled

The marriage slid downhill with Om entering the *apāyamuk*, being physically violent with Chhaya and having multiple relationships with other women, to the point that Chhaya fled to Phnom Penh, taking their daughter with her. Increasingly dissolute, Om reached the point where his physical health was ruined, and he had what appeared to be a stroke. When he died soon after, Chhaya had to fulfill her obligation by participating in his funeral and staying for the 7-day funeral ceremony. By now, the significance of the series of omens had sunk in. The arrival of the snake, the “peril animal,” had followed on the heels of the “peril woman” /srəy /, and tragedy had indeed befallen her family.

### Power and Money

Huge disparities of wealth and power create the scene for exploitation, victimization, and acid attack. One scenario is that a politically powerful and high-ranking married man takes control of a lowly and vulnerable young woman, with his jealous wife often hiring thugs to do the dirty work. Among the highest-profile cases was that of 16-year-old Tat Marina in 1999, attacked in broad daylight, allegedly by the wife of a high-ranking government official, with no arrests ever being made.

Orn Soniya, aged 23, had been seduced by Heng Hok, a policeman connected to the political party in power, who lied that he was divorced. They started living together and she became pregnant. His wife Sokha tracked them down in a far-away province and hurled acid at her. Soniya compared her life journey to that of the maiden Sumantie as told in a folk tale “The legend of Prince Sang Sel Chey,” Saing Silachey, /rɨəŋ saŋ səl cey/ (រឿងស័ង្ខសិល្បជ័យ), which was published by the Buddhist Institute (Uknha Vangsa Dhipati Ouk, 1966) and which is a Khmer rendition of a legend widely known in Southeast Asia as “The Prince of the Golden Conch Shell,” inspired by the Paññāsa Jātaka. One version goes like this. The royal astrologer predicted that the King’s sister Somantie had bad *graha* and as a result would end up far away from the kingdom. To forestall this, the king prevented her from wandering away. However, 1 day she managed to visit the forest and there a Yakkha ogre who had transformed himself into a prince fell in love with her and carried her away to his kingdom, where they lived together. Even after she had discovered his true identity, she loved him, but she was predestined to be parted from her love, as the king wanted to bring her back to the palace. Saing Silachey came to take her back to the castle and, as they were on their way, the Yakkha ogre awoke and tried to stop them, but he was killed by an arrow. In the end, Somantie suffered twice, first as a result of the abduction that led to the loss of her royal life and her family kingdom, and then by widowhood from her beloved.

The tale contains some familiar themes, such as falling for a person who is not what he seems. A nubile beauty like Somantie, Soniya was seduced by the policeman Hok, a “big guy,” a sort of earthly political giant. Like Somantie, who was deceived by a giant Yakkha ogre masquerading as a human, Soniya was seduced by the married policeman pretending that he was divorced. Like Somantie, she was taken away from her family and had a baby with her giant. Like Somantie, who was tracked down, she is tracked down by her giant’s wife, Sokha. Like Somantie, whose loving relationship with her giant Yakkha was rudely severed when her relations killed him, leaving her in a state of grief, Soniya experienced lifelong grief over the loss of her beauty and her future when Sokha threw acid on her. Like Somantie, who was astrologically incompatible with her giant, Soniya was incompatible with her political “big man.” Each of the two, Soniya and Sumantie, had bad astrological *graha*, as was manifested in their inappropriate love relationship, each of which was predestined to disaster.

Disparities of power and wealth can operate in the other direction too. Moeun Sean, 22, was an intelligent and attractive woman but her family lacked the funds to bribe her entry into higher education, and she used her considerable talents and intelligence to embark on a successful career as a corn wholesaler. Ming Socheat, 32, one of her employees, was unhappily married to Tem Danaet with whom he had several children, and they were in the early stages of an amicable divorce. At first, Sean felt nothing but pity for this poor, illiterate, and rough man who was so socially and economically beneath her, but somehow, she found herself falling in love with him, and she spurned the advances of other, far more eligible men. She said he must have resorted to casting a love charm on her which, she thought, he had put into a bottle of fancy imported perfume. Soon enough, she said, it took full effect, and, spurning the attention of the other suitors, she surrendered. When Socheat’s wife discovered the affair, she halted the divorce proceedings to block Socheat from being able to legally marry Sean. One night, Sean dreamt that she was seated on her high throne when, all of a sudden, the ritual paraphernalia surrounding her throne fell to the ground, which she took to be a warning omen from her spiritual master-teacher. Shortly thereafter, Socheat’s wife got her accomplices to hurl a bucket of acid at Sean; she lost both her eyes, was severely disfigured, and lost her wealth—and her lover.

Sean likened her life to that of a character in a popular movie from the Golden Age of Khmer cinema entitled “Tep Sodachan” (ទិព្វសូដាច័ន្ទ). Produced in 1968, on the eve of the Khmer Rouge coming to power, it is still widely circulated on media today. The movie is loosely based on a historical folktale about the mythical origin of the construction of Angkor Wat temple. The goddess Sodochan lived in the land of the deities, ruled by her father Indra, King of the deities. Gazing down upon the earth, she saw the impoverished peasant Veasna, whose mother had died of misery and because of the cruelty of a rich moneylender, and who had been forced into debt bondage with the moneylender. Feeling pity for him, she asked permission to descend to the earth to help Veasna. Transforming herself into a poor young woman, she asked Veasna if she could shelter in his house, and Veasna allowed her to stay inside while he slept outdoors. Despite their massive cosmic, social, and economic differences, the goddess Sodachan fell in love with the human Veasna and, aided by a guardian spirit, they joined up to create a loving home “beautiful by the moonlight” and had a child. The story ends with grief and sorrow, for Sodachan lost Veasna. The movie remains a compelling story in contemporary social media, telling of the impossible dreams of love across socio-economic gaps—Sean’s bitter experience is that love can bloom between the rich and the poor, but all that can be destroyed by the acid attack of a jealous wife. As graphically foretold in her dream, she plunged from her throne into misery and squalor.

Sean’s tragic story shows how jealousy can operate across the economic divide in a love triangle. Economics also plays a role in setting the scene for the attack in the context of IPV, with men spiraling down the cycle of poverty, seeking refuge in heavy drinking and gambling. Marriages fail and reconciliation becomes unlikely, driving the rejected spouse to retaliate. One further emerging aspect is the small number of cases of acid attacks affecting gay and lesbian relationships, “third gender love” /snaehaa pʰeet tii bəy/ /(ហាភេទទីបី). Such individuals are embroiled in what is culturally viewed as “unnatural” sexual relationships and, (judging purely from my own personal experience), such cases are primarily associated with wealthier families in Phnom Penh.

Attacks lead to socioeconomic consequences. Ty Vy, a survivor of an attack, depicted her life, plunged into eternal poverty, as akin to “a life battlefield,” /saʔmaʔraʔpʰuum ciivɨt/ (សមរភូមិជីវិត). However, while even wars come to an end, her battlefield is endless and lifelong.

**Figure 4.**
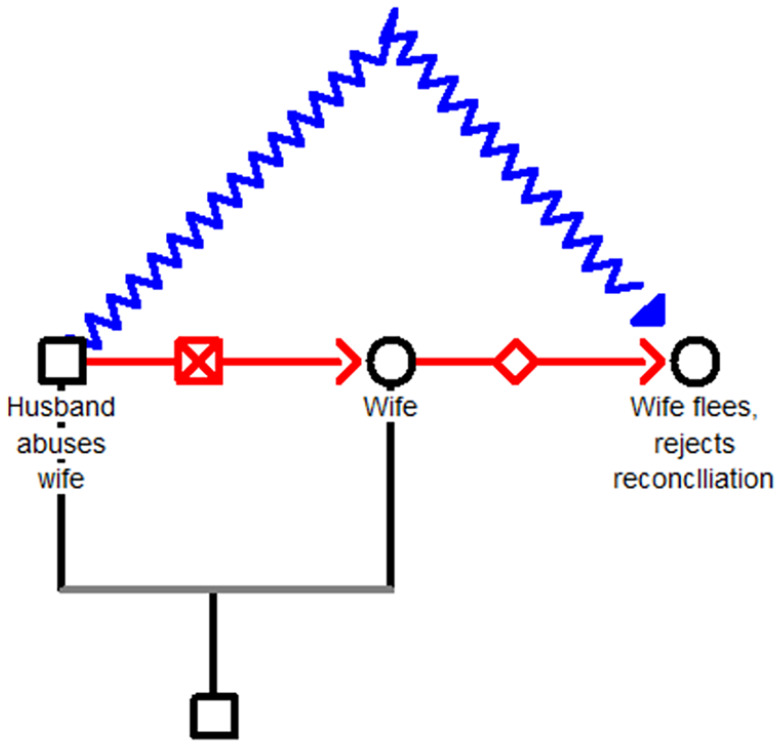
The wife flees the abusive husband and rejects his efforts at reconciliation. In response, he hurls acid at her.

During the COVID-19 pandemic, cases involving triangular love seemed to dry up, and the only cases that persisted were those involving IPV. In this second taxon, there is no third party. The problem evolves from the husband’s physical and emotional abuse of his wife. There are two scenarios based on whether the wife can flee. If the wife manages to flee her abusive husband and rejects his efforts at reconciliation and pulling her back under his control, his response is to hurl acid at her (Figure 4).

**Figure 5.**
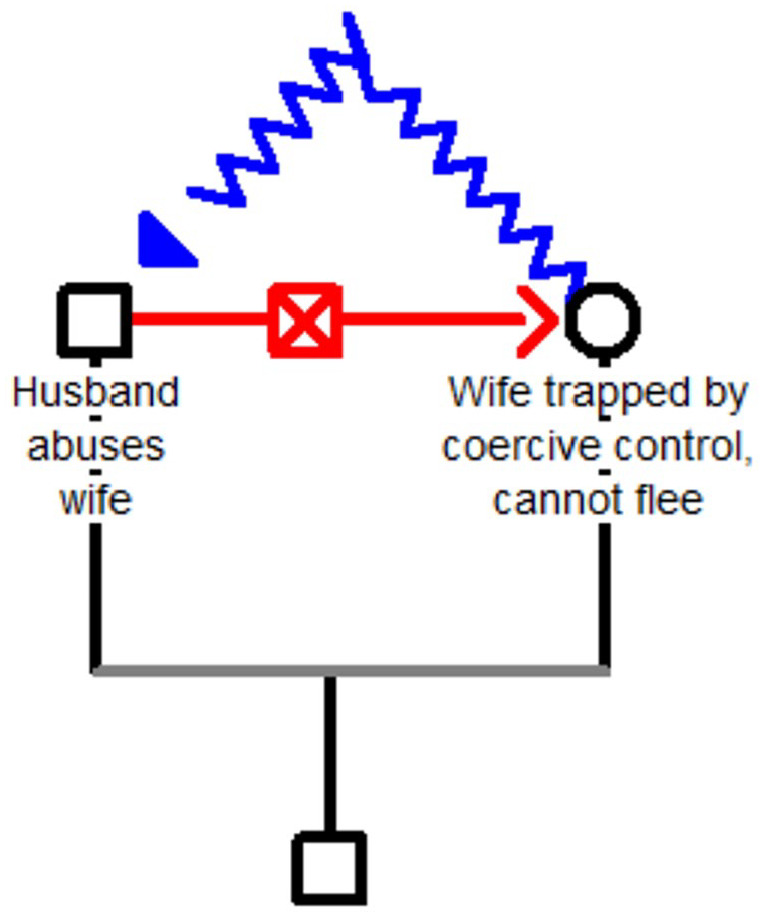
The abusive husband exerts coercive control and prevents his wife from escaping. She hurls acid at him.

Sometimes there was a background of triangular love here too, when estranged husbands who had abandoned their families for other women, but were now jobless and out of money, had lost their value to their girlfriends and crawled back to their first wives. When, given the circumstances, their wives closed the door in their faces, they retaliated by perpetrating acid attacks. Some women who are economically dependent on their husbands and vulnerable to coercive control cannot flee easily; eventually, some are driven in desperation to throw acid at their husbands (Figure 5).

## Discussion

An acid attack is an expression of burning emotions leading to the burning of people. The burning emotions here are romantic jealousy and envy and anthropologists have long been interested in the jealousy/envy syndrome. Exploration of the meaning of the emotional drivers of acid attacks leads to the elucidation of the cultural context of complex emotions of jealousy and envy.

Romantic jealousy is present across all societies, and there is evidence that it is culturally constructed (Hupka et al., 1985; Zandbergen & Brown, 2015). Jealousy is commonly thought of as a combination of possessiveness and extreme anger, but how are these two emotions experienced and expressed in cultural contexts? Cross-cultural studies are necessary to obtain a clearer picture of the triggers of GBV and other forms of violence in men and women. For example, according to Warrell (1990), Sinhalese Buddhists in Sri Lanka describe themselves as jealous people (*okoma* and *serama irisiava*) and consider jealousy the basis of their culture. The findings here direct our attention to Buddhist theory on the fire of anger and jealousy, as expressed, for example, in the Gattva Suta. The association between anger and heat has deep historical and cultural roots in the Hindu religion (Lange, 2022), while Dilani (2022) points out that sexual jealousy is found as a theme in several Jātaka such as the Chaddanta Jātaka (No. 514), the Culla-paduma (No. 193), and Mahā-Padhuma Jātaka (No. 472).

The first taxon of the acid attack, triangular love, is driven by romantic jealousy, which, in Cambodia, is metaphorically conceptualized as fire and literally known as “the fire of jealousy,” /pləəŋ prɑcan/ (ភ្លើងប្រច័ណ្ឌ), derived from a prefix /pra-/, meaning “in excess, retrospective,” and the root /can/, from Sanskrit and Pāli *caṇḍa*, meaning “fierce, violent, uncontrolled.” In the Agati Sutta, the Off-Course Sutta, it is one of the four main emotions of violence.

Another angle on Buddhist teachings is evident in informants’ beliefs regarding the origins of the emotions of anger and possessive jealousy, and “the fire of revenge.” The case of Cheng and Reaksmy demonstrates how smoldering feelings of revenge are a problem, especially when, as A. L.Hinton (1998) noted, there is a tendency among Cambodians towards “a head for an eye,” or disproportionate revenge, as so tragically exemplified in acid attacks.

One can see the possessive quality of romantic jealousy and the lengths to which a person can go. The anguish can be so intense that, at least in some instances, the perpetrator can no longer face life and would rather be “reunited” with his ex-lover by drinking the same acid he had used to destroy her.

Envy burns strongly in a country like Cambodia where there are so many in need, and it is a “social emotion because it is nested in social comparison” (Quintanilla & de López, 2013, p. 82). Being envied, rather than being a source of prestige, is dangerous as it leads to many crimes, including assassination and acid attacks. Envy involves two people, and jealousy involves three: “an envious person lacks, or at least believes that he lacks, the object, whereas a jealous person possesses, or at least believes that he possesses, the object and hence is afraid to lose it” (Quintanilla & de López, 2013, p. 78). These insights can be applied in fields such as transcultural psychiatry, extending the seminal work on morbid jealousy; for example, by Buddhist psychologists like De Silva and De Silva (1999) in Sri Lanka, which, like Cambodia, is a Theravada Buddhist society. Envy in Khmer is /crɑnaen/ (ច្រណែន), the near-synonyms are /cnie niih/ (ឈ្នានីស) and /rɨhsyaa/ (), the last borrowed from Pāli *issā*, one of the unwholesome mental factors (*akusala-cetasika*) which, together with greed (*lobha*) and avarice (maccariya) involves attachment to an object (Goleman, 1975) and is a cause of conflict (Lwin, 2016). Envy leads the perpetrator to a state of “moral blindness,” a state of mind in which they perpetrate the acid attack.

### Cultural Attractors

Even though the human rights literature on acid attacks strongly defend women as the predominant targets, the cultural drivers, at least in South and Southeast Asia, are problematic in that women tend to be blamed. Popular views about the cultural drivers of acid attacks are heavily drawn from stereotypes and culturally popular beliefs about men and women. For example, men who create love triangles seem to have a cultural escape clause in being able to blame supernatural markers such as the penile mole for ineluctably lassoing multiple women with impunity (Eisenbruch, 2018a), thereby generating the heat of romantic jealousy in triangular love.

Further, there is a view that the popular beliefs described here are related to Cambodian readings (perhaps misreadings) of Buddhist teachings. In terms of endowment, for example, the reported blaming of the woman seemingly has roots in Cambodian Buddhism, as in the Sujāta Jātaka, (7:59; IV 91–94) (SuttaCentral et al., 2017), in which the Buddha chides an arrogant wife and, indeed, the suggestion has been made that the Jātaka stories tend to portray women as vicious (Roy, 2023).

The descriptions by the informants referenced a range of defects of character and moral conduct by the perpetrator and, often, by the survivor, such as the “three unwholesome roots”—the *akusala-mūla*, comprising ignorance, attachment, and aversion; these are important in understanding acts of impunity in Cambodia (Eisenbruch, 2018a).

In popular usage in Cambodia, /krʊəh/ (គ្រោះ), or *graha* in Pāli, means an individual’s astrological good or bad fortune, and when coupled with the term for peril, /cɑŋray/ (ចង្រៃ), means misfortune or mishap (Eisenbruch, 2020), a product of the person’s astrological destiny coupled with their own conduct.

As for what goes on between a couple, astrological incompatibility is used as a popular explanation for domestic violence, including acid attacks. The “Sɑmpʊəŋ Manual” (សម្ពង្ស), (the Khmer word is derived from the Pāli *Vaṃsa*, meaning “lineage”) is based on an astrological theory that determines whether a man and a woman will make a good couple based upon the month and year of their births.

It could be assumed that acid attacks are, by definition, premeditated in that people do not just carry acid around with them, and therefore, there is always an opportunity to reconsider. The popular belief, however, is that the frenzied perpetrator is in a temporary state of insanity. This is described by our informants in a Buddhist idiom as a temporary state of “moral blindness”—literally, “closed and turned off by *moha*, cloaked by *moha*” /moo bət moo baŋ/ (មោហ៍បិទមោហ៍បាំង), from the Pāli *moha* for illusion, delusion, or emptiness, and the reduplication of the word in the phrase intensifies the meaning to total and absolute moral blindness.

### The Love Triangle

The love triangle is ubiquitous in history and found in the classical literature of Japan (Kuchta & Malita, 2015), China (Chen, 2010), and Thailand (Barua, 2017). Cambodian literature furnishes many stories involving love triangles and they have been a popular motif in the arts. In his film *Twilight*, for example, the late King Norodom Sihanouk starred as the Khmer Prince Adit in the tale of a love triangle, a theme also found in folk tales such as Phadaeng Nang Ai in Laos and Thailand. In romantic jealousy, there is a threat to the relationship in which a person perceives a real or imaginary attraction between their partner and a rival (Aloyce et al., 2023; Martínez-León et al., 2017; Pichon et al., 2020). Acid attacks in various Asian countries are often attributed to love triangles. In Buddhist teaching, the Agati Sutta (the “Off-Course Sutta”) and the Parābhava Sutta (the “Discourse on Downfall”) are meant to deter people from entering the Road to Ruin (*apāyamuk*). The love triangle is clearly associated with jealousy, an emotion which, according to the Parābhava Sutta, leads to downfall (Jayawardena, 2019). This fire burns in the perpetrator’s heart, consuming them with jealous rage and driving them to commit the acid attack—ironically, burning the target of their jealousy.

### Love and Love Magic

Lovesickness in various Southeast Asian societies is popularly believed to be induced by a magical love charm, as seen in Laos (Westermeyer, 1979) and Thailand (Rajadhon, 1964; Somchintana, 1979). There are two types of love charm in Cambodia: the first is “love charm to destroy and separate” /snae bɑmbaek/ (ស្នេហ៍បំបែក), to break the husband’s bond with his wife. The second has two complementary functions: to drive him crazy with lust for her, literally a “love charm to arouse and entwine,” /snae prɑteʔpoat (ប្រតិព័ទ្ធ) and, ultimately, to induce him to fall in love with her, literally, “love charm to make the heart tender,” /snae bɑntʊən cət/ (ស្នេហ៍បន្ទន់ចិត្ត). A new finding that arises from our study is the role of the love charm in breaking up a marriage by forcing the creation of a love triangle and, thence, leading to acid attack. The case of Ky Phea, who hurled acid at Sean Chany, involved his exploiting his dental love charm, which is similar to a technique I have previously reported utilizing a Yantra known as the Precious Crystal Canine Tooth of the Buddha, /preah cɑŋkoom kaev*/* (ចង្កូមព្រះកែវ), literally drilled by the dentist into the love-charmer’s canine tooth (Eisenbruch et al., 2013). This technique was rumored to have been used by Phea to ensnare Chany. Acid attacks in the context of triangular love share a foundation with “lovesickness,” /snae/ (ស្នេហ៍) or, in its more extreme form, “madness of lovesickness,” /ckuət snae/ (ឆ្កួតស្នេហ៍), brought about by a magical love charm cast by a rival (Eisenbruch, 2010).

### Changes

A further issue for consideration is what changes have occurred in acid attacks over the years. It was hoped that community education and the introduction of acid legislation would curb the prevalence of attacks but, despite claims by the authorities, the weak impact of such laws has been highlighted in countries such as India and Bangladesh (Ahmad, 2012; Shrikanthan et al., 2019; Taylor, 2000).

One unfortunate issue is that attacks continue to go unreported, for example, in India (Hameed & Bhattacharya, 2022), and legal measures to bring perpetrators to justice have not been entirely successful in countries such as Bangladesh (Calcini, 2022), Cambodia, (Calcini, 2022; Son Minea, 2022), Colombia (Calcini, 2022), Egypt (Shehata et al., 2022), India (Ahmad, 2012; Calcini, 2022), and Pakistan (Bibi et al., 2021; Calcini, 2022). Indeed, recent reports show attacks to be continuing (Rossoni & Campofreda, 2023), with a reported rise in AV becoming a global issue and 1,500 cases documented annually (Ahmed et al., 2017).

In Cambodia, it has been claimed that after the promulgation of the 2012 Acid Laws, the frequency of attacks was reduced. As also discussed earlier, my personal experience of what happened in Cambodia is mixed. True, it became more difficult to obtain the strongest “acid, number 1” and attacks were more likely to be perpetrated using “acid number 2,” that is battery acid. Perpetrators were more likely to be imprisoned. Survivors gained greater access to support services and, in theory, had the right to compensation decreed by the court. However, the perpetrators usually evaded having to pay compensation and, in terms of emotional and cultural dimensions, there was no change in the basic drivers of acid attack, that is, anger, jealousy, and envy.

### Challenges and Limitations

Challenges of researcher positionality are well-noted in research on acid attacks (Jabeen et al., 2021; Rahbari, 2022). The encounters with survivors of acid attack were among the most challenging in the author’s experience as a clinician and ethnographer, dedicated to helping people who are suffering but incapable of meaningfully offering help beyond having created a safe space to listen attentively to the survivor’s story. Even after decades of experience as an ethnographer in Cambodia, it was difficult to deal with one’s own reactions to “culturally embedded” gender violence, and awareness of a wish to advocate on behalf of the survivors. The female Cambodian research assistant (CP), herself a Buddhist who adhered to teachings about karma, coped with the trauma of witnessing such disfigurement and suffering among fellow women by eliciting the informants’ stated explanations, such as their karmic predestiny from their previous life, and showing acceptance that, even if CP did not fully believe this logic, this was true *for them* and, as such, was a source of comfort. Therefore, the research objective of eliciting the informants’ views also helped the team to cope.

Another potential limitation is that the findings presented here are taken as essentialist, as if culture shaped gender violence. Intersectionality is important in research on acid attack (Mittal et al., 2023) and I do not wish to privilege the cultural over social, economic, structural, and feminist dimensions.

## Conclusion

This study, focusing on Cambodia, sheds light on the cultural drivers and meanings associated with acid attacks. The study shows how important it is to view a phenomenon such as acid attack as more than one thing—here, three distinct taxa, each driven by different cultural dynamics. The first revolves around romantic jealousy within a love triangle, where the metaphorical “fire of jealousy” consumes the perpetrator and leads to the attack. Love charms are believed to play a role in initiating some of these love triangles, pulling individuals into romantic entanglements that they may not have chosen willingly. The documentation of such beliefs and practices enables a greater understanding of a person’s lack of agency and the ascription of blame. The second taxon involves IPV, where abusive husbands, seeking to maintain control over their wives, resort to acid. Finally, the third taxon is characterized by broader social and political conflicts, where disputes among peer groups, envy, and economic tensions escalate to acid attacks.

This study underscored the importance of considering cultural elements that are believed to exert coercive control over women. These include beliefs in karma; for example, where survivors may be made to feel responsible for their own suffering because of their past actions. However, this study suggests a more nuanced understanding, where the perpetrator and survivor share a mutual karmic entanglement that can potentially guide them toward avoiding suffering in the future. Drawing from Buddhist psychology, the study identifies the Triple Poisons of ignorance, attachment, and aversion as factors that fuel acid attacks. In cases of romantic jealousy, the “slow fire of revenge” is associated with *lobha* (sexual desire) and *dosa* (destructive anger).

Intersectoral understandings of GBV take us beyond essentialist approaches that privilege economic, political, or cultural factors alone. The popular view, especially in South Asian countries such as India, is that acid attack is a form of GBV, but the various accounts given seem to suggest that gender is not necessarily the driving force in many of the attacks; the person attacked is the “rival” or the “betrayer,” male or female, in a given situation. In a significant number of the cases here, gender does not have a role, other than the fact that the majority of relationships involve a man and a woman, and that only applies to the cases related to jealousy: cases involving envy do not seem to be gender-related at all. While individual cases of acid attack may not necessarily be due to the desire to target a specific gender, such attacks are generally considered to form part of the spectrum of GBV, along with IPV and other forms of violence. Given that the attacks described can be by men on women, women on men, women on women, and men on men, and do not seem to be carried out specifically because of the target’s gender but rather because of their role in the particular situation that prompted the attack, more work is needed to clarify the nature of the culture-gender intersect.

There are broader anthropological and psychological implications for the cross-cultural understanding of complex emotions such as envy and romantic jealousy as drivers of possessive or vindictive behavior. Romantic jealousy is metaphorically represented as a consuming fire in Cambodia. Envy, exacerbated by social inequality, is a driver of community conflicts that easily escalate into direct and public violence. The findings on the expression of emotions in acid attacks—not only the anger of the perpetrator and, for that matter, the survivor, but the other emotions that can come into play, such as sexual attraction and love, fear, and the complex emotions such as jealousy and envy—also apply in other settings of violence, for example, domestic violence, child sexual abuse, monastic abuse, public violence, and suicide. These emotions seem to be embodied and expressed in terms of heat and fire.

The cultural context contributes to understanding economic factors, especially those arising from poverty and financial issues, and how economic stressors can be a catalyst for GBV.

The provision of mental health, rehabilitation, and psychosocial support and care for survivors could be enriched by a greater understanding of “Buddhist trauma therapy,” in which a culturally coherent psycho-education (for example, suttas to which ordinary people can easily relate, along with the performance of healing rituals) can help in the management of complex and destructive emotions such as anger and vengeance. This way, the risk of suicide could be lessened as survivors (and perpetrators) become remoralized.
